# Cysteinyl-tRNA Synthetase 1 Promotes Ferroptosis-Induced Cell Death via Regulating GPX4 Expression

**DOI:** 10.1155/2022/4849174

**Published:** 2022-09-28

**Authors:** Wanfei Zhang, Xianzuan Lin, Shaogeng Chen

**Affiliations:** ^1^Quanzhou First Hospital, Department of Thoracic Surgery, Fujian, Quanzhou, China; ^2^Quanzhou First Hospital Affiliated to Fujian Medical University, Fujian, Quanzhou, China

## Abstract

Esophageal squamous cell carcinoma (ESCC) has still been considered to be the most common malignant tumors in China. Emerging evidence indicates that cysteinyl-tRNA synthetase 1 (CARS1) has been considered as a ferroptosis-related gene in ESCC. However, the roles and molecular mechanisms of CARS1 in ferroptosis-induced cell death of ESCC are still largely unknown. In our study, we investigated an aberrantly upregulated gene in ESCC tumor tissues CARS1 significantly inhibited cell proliferation, and the ability of migration and invasion promoted the relative level of MDA and ROS and decreased GPX4 expression level in two ESCC cell lines. Mechanistically, both the ferroptosis inhibitor ferrostatin-1 and its inducer erastin were further used and indicated that CARS1 participated in the ferroptosis-induced cell death. Together, these results revealed that CARS1 has a critical function in the progression of ESCC by promoting ferroptosis-induced cell death.

## 1. Introduction

Despite the development and progress in diagnosis and therapy, esophageal cancer is still considered as a common malignant tumor worldwide. Esophageal cancer is a digestive system tumor and could mainly be classified as two pathological types. One is esophageal adenocarcinoma (EAC) which is the most common in the developed countries, and the other one is esophageal squamous cell carcinoma (ESCC) which is the main pathological tumor with a high mortality and poor prognosis and has become the main threat to human health in China [1, 2]. Therapy for the early diagnosis of ESCC is still limited, and thus, most people lose the opportunity of surgical therapy. Due to the difficulties in the early diagnosis of ESCC and poor prognosis for ESCC patients, it is important to identify the critical gene in the development of ESCC and reveal its regulatory mechanism. The novel targets would be useful for the therapy of ESCC treatment.

Recently, a novel identified biological process of cell death has been investigated, which is called ferroptosis and caused by iron-dependent lipid peroxide accumulation [3]. Recent evidence has suggested that ferroptosis is involved in various pathological processes, for example ischemia-reperfusion injury, cancer cell death, and neurotoxicity [3, 4, 5]. Ferroptosis activation would lead to nonapoptotic destruction in several cancers, and the suppression of one of the processes may protect organisms from neurodegeneration. The regulation of ferroptosis is a complex regulatory process, mainly regulating genes such as free iron ions and lipid peroxidation metabolism. Its main feature is the accumulation of iron ions in cells, which requires lipid peroxidation. Ferroptosis is mainly manifested in the increase of ROS accumulation and glutathione and a diminished activity and content of glutathione peroxidase 4 (GPX4) [6, 7]. It would induce the cells to exhibit a smaller mitochondrial volume, a reduced number of ridges, and an increased density of membrane. Importantly, GPX4 has an important effect on ferroptosis inhibition and could convert lipid peroxides into inert lipid alcohols. Therefore, the inducer of ferroptosis would have a strong effect on the inhibition of tumor growth and may become a new attention for novel therapy in tumor treatment in recent years.

Cysteinyl-tRNA synthetase 1 (CARS1) belongs to a class 1 aminoacyl-tRNA synthetase and contains one of several posited around the imprinted gene domain, which is known as a critical gene region, and inhibited tumor growth. Recent research has indicated that CARS1 is close to ferroptosis and has been predicted as a prognostic factor [8, 9]. Therefore, to explore the biological role of CARS1 in ESCC and further clarify the molecular biological mechanism of ESCC and provide a new biomarker for ESCC therapy is essential.

## 2. Materials and Methods

### 2.1. Clinical Samples

In the study, 60 patients were collected who had been diagnosed with ESCC and had received a surgical resection. These tissue samples have been further confirmed pathologically after resection.

### 2.2. Cell Culture and Transfection

In the current study, KYSE-30 and KYSE-410, two human ESCC cell lines, were acquired from the cell bank of the Chinese Academy of Sciences. The 1640 medium (Gibco of Thermo Fisher Scientific, Grand Island, NY) has been applied for cell culture with the addition of 10% FBS in a 37°C container with 5% CO_2_. The Lipofectamine 3000 reagent has been applied for transfection. The CARS1 gene was synthesized and cloned into plasmid pCDH-CMV-MCS-EF1-copGFP-Puro. GFP was used to check the transfection efficiency. Seed plate: the day before the transfection operation, cells with appropriate density were inoculated into the six-well plate. Fluid change: before the transfection operation, discard the old medium in the six-well plate and replace it with 2 ml of fresh medium with serum but no antibiotics. Transfection: prepare at least twice the amount of sterile 1.5 ml EP tubes in advance according to the number of holes in the six-well plate. For each well of cells, add 125 *μ* L fresh DMEM culture medium (without adding antibiotics and serum) to tube A and tube B, respectively; the amount of substance added in tube A is 2.5 *μ* G of plasmid DNA and 5 *µ*l of auxiliary transfection reagent p3000 Gently blow and suck them with a pipette to mix them. Then, add 5 *µ* L to tube B of lipo6000 transfection reagent. DMEM and lipo6000 were also blown and mixed and incubated at room temperature for five minutes. Then, the solution in tube A added with plasmid DNA was sucked into the mixed solution containing transfection reagent in tube B, the two tubes of solution were blown and mixed with a pipette, and the mixed solution was added to the six-well plate after standing at room temperature for 20 minutes. Place the six-well plate in the incubator for 24–48 h and change the fresh medium every 8–12 h after transfection. After 24–48 hours of transfection, the overexpression efficiency was verified and other subsequent experiments were carried out. Following the design and synthesis of shRNA-CARS1, the transfection was carried out.

### 2.3. RT-qPCR Assay

Total RNA has been prepared from tissues or cell transfection and then synthesized into cDNA. The internal reference was the GADPH, and the CARS1 level has been further examined by the 2^−ΔΔCT^ method.

### 2.4. Cell Proliferation Assay

For the colony formation assay, a density of 1 × 10^3^ transfected ESCC cells has been cultured into a six-well plate. After two weeks of culture, the clones have been further stained with 0.1% crystal violet. For CCK-8 assay, a density of 2 × 10^3^ transfected cells have been cultured in a 96-well plate. Later, the old medium was exchanged with a fresh medium containing 10% CCK-8 reagent. A wavelength of 450 nm was used for the measurement.

### 2.5. Flow Cytometry Detection

The treated cells were resuspended with the binding buffer. The final density was 1 × 10^6^ cells per milliliter. Dilute DCFH-DA with serum-free medium at a ratio of 1:1000 to make the final concentration of 10 *μ* mol/L. After collection, the cells were suspended in diluted DCFH-DA with a cell concentration of 1 million to 20 million cells/ml and incubated in a 37°C cell incubator for 20 minutes. Mix it upside down every 3–5 minutes to make the probe fully contact the cell. The cells are washed with serum-free cell culture solution three times to fully remove DCFH-DA that does not enter the cells. After a thorough mixing, the suspension was immediately detected by the FACScan flow cytometer system.

### 2.6. Cell Invasion Assay

The Corning Biocoat Matrigel Invasion Chambers were performed to measure the ability of cell invasion. The density of 1 × 10^5^ treated cells per well was suspended in the serum-free medium under the upper chambers. The lower chamber was injected with the normal medium as a chemoattractant. After a certain time, the top transwell containing the noninvaded cells was discarded, while the bottom transwell containing the invaded cells was fixed and then stained for another 15 min.

### 2.7. Cell Migration Assay

The ability of migration was used as described for the invasion assay except that Matrigel was not injected and treated cells were cultured for an additional 16 h.

### 2.8. MDA Assay

The lipid peroxidation assay kit (ab118970, Abcam, MA) has been used to examine the concentration of relative malondialdehyde (MDA) following the manufacturer's protocol. In brief, thiobarbituric acid (TBA) was injected into the sample, was made to react to the MDA-TBA adduct, and then was measured colorimetrically.

### 2.9. Western Blot

Total protein has been prepared and then loaded on the SDS-PAGE gel electrophoresis. Subsequently, protein from the gel was transferred to the membrane and then incubated with 5% skimmed milk for blocking. The prepared-membrane was then incubated with the primary antibody. In another day, these membranes have been treated with the secondary antibody. Finally, the membrane was detected by the chemiluminescent HRP substrate.

### 2.10. Statistics

The SPSS 20.0 has been applied with the Student's *t*-test and one-way ANOVA have been utilized to examine the difference. A *p* value lower than 0.05 has been considered significant statistically.

## 3. Results

### 3.1. The Relative Level of CARS1 in ESCCs

**Figure 1:**
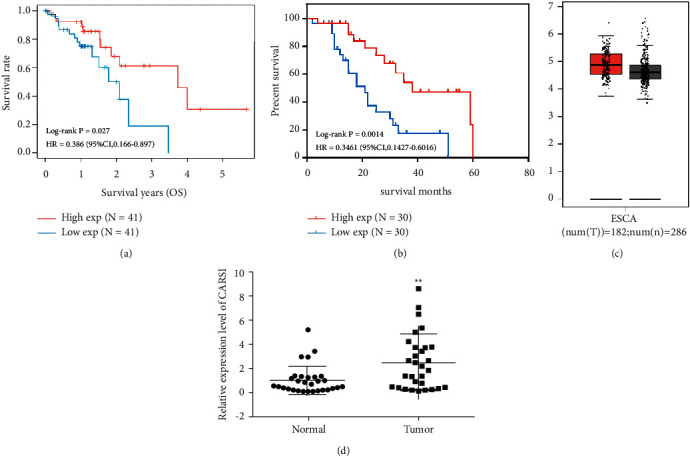
Identification of CARS1 level in ESCC. (a) Data from the TCGA database and collected ESCC patients (b) revealed the that lower CARS1 expression is correlated with poor overall survival in ESCC patients. (c) The relative expression of CARS1 in ESCC (*n* = 60) and normal tissues (*n* = 60) from GEPIA database. (d) The relative expression of CARS1 in ESCC tissues and adjacent normal tissues collected. ^*∗*^*P* < 0.05 and ^*∗∗*^*P* < 0.01.

Based on the ESCC data from the TCGA cancer transcriptome database, CARS1 was remarkably associated with the prognosis (*P* = 0.027). The higher expression of CARS1 could increase the survival ratio of ESCC patients (Figure 1(a) and 1(b)). The GEPIA database indicated that the relative level of CARS1 in ESCC patients exhibited an increase to the normal tissue (Figure 1(c)). Subsequently, we collected 60 ESCC patients and analyzed the clinical data and the postoperative pathological reports. It suggested that the CARS1 mRNA level exhibited an increase in ESCC tissues than in normal esophageal mucosa tissue (Figure 1(d)).

### 3.2. The Role of CARS1 in ESCCs

**Figure 2:**
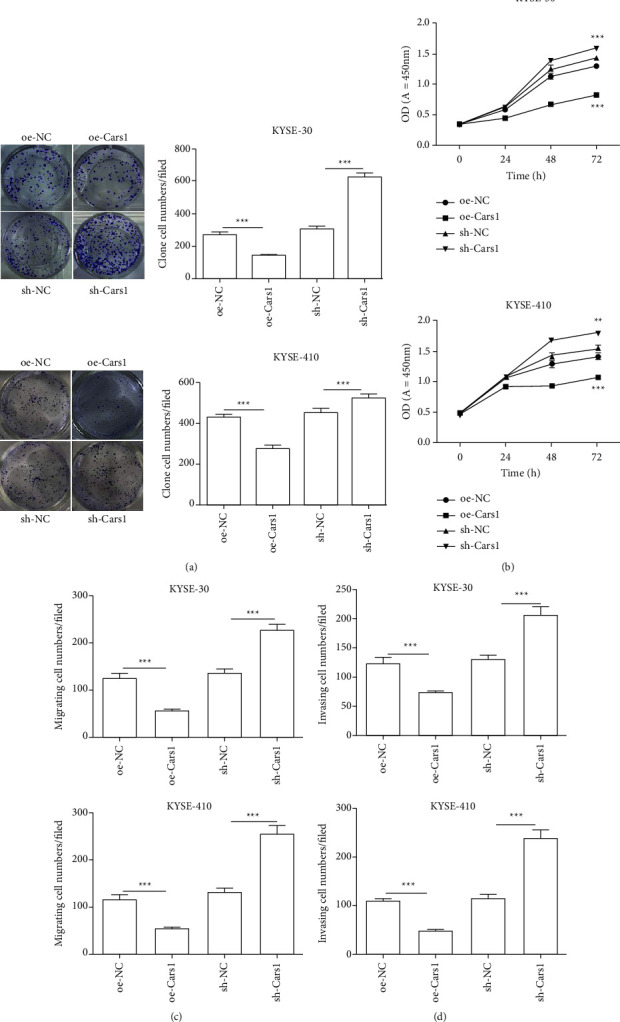
Effect of CARS1 on ESCC cell proliferation and the ability of migration and invasion. The colony formation assays (a) and CCK-8 assays (b) were measured after transfection, respectively. The migration (c) and invasion (d) abilities in KYSE-30/410 cells after transfecting have been examined by transwell assay, respectively. ^*∗*^, ^*∗∗*^, ^*∗∗∗*^ indicate *P* < 0.05, 0.01, and 0.001 respectively.

To explore the role of CARS in ESCCs, we first upregulated and downregulated CARS1 in KYSE-30 and KYSE-410 (Figure S1). The results showed that upregulated CARS1 decreased the colony formation count as compared with the control group in ESCC cell lines. However, downregulated CARS1 increased the colony formation count as compared to the control group (Figure 2(a)). Next, we measured cell proliferation by the CCK-8 assay. It indicated that upregulated CARS1 inhibited the viability more than the control group at 72 hrs in KYSE-30 and KYSE-410, and downregulated CARS1 promoted the ability of proliferation at 72 hrs as compared with the control group (Figure 2(b)). The colony formation assay and the CCK-8 assay suggested that upregulated CARS1 would inhibit the proliferation of ESCCs and downregulated CARS1 would promote the proliferation. Further, we analyzed the migratory and invasive abilities of two cell lines. Based on Figures 2(c) and 2(d), upregulated CARS1 inhibited cell migratory ability and downregulated CARS1 promoted invasive ability. Taken together, these results suggest that CARS1 has an inhibitory function on proliferation, migration, and invasion in ESCCs.

### 3.3. The Effects of CARS1 on Redox Reaction in ESCCs

**Figure 3:**
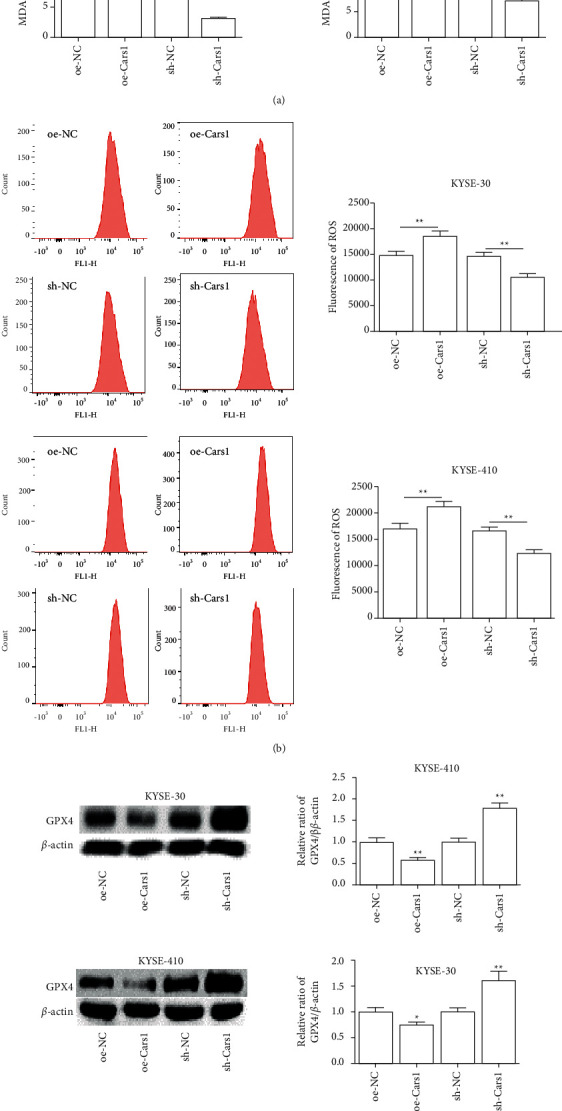
Effect of CARS1 on ESCC cell redox reaction. (a) The relative level of MDA is measured in KYSE-30/410 cell transfection. (b) Flow cytometry is conducted to determine the ROS content in KYSE-30/410 cells after transfecting. (c) Western blot assay is further performed. ^*∗*^, ^*∗∗*^, ^*∗∗∗*^ indicate *P* < 0.05, 0.01, and 0.001, respectively.

The MDA concentration ESCCs were further examined with the lipid peroxidation assay. It indicated that upregulated CARS1 increased the relative level of MDA as compared with the control group and downregulated CARS1 decreased the relative concentration of MDA in KYSE-30 and KYSE-410 (Figure 3(a)). Subsequently, the flow cytometry assay indicated that upregulated CARS1 promoted the content of ROS compared with the control group and downregulated CARS1 inhibited it (Figure 3(b)). Additionally, Western blot indicated that GPX4 expression level significantly reduced in KYSE-30 and KYSE-410 transfected with CARS1, and downregulated CARS1 had an opposite effect (Figure 3(c)). Accordingly, it suggested that CARS1 activated the redox response in ESCCs and had an inhibitory effect on the expression of GPX4.

#### 3.3.1. The Effects of Upregulated CARS1 and Ferrostatin-1 on Cell Proliferation and the Ability of Migration and Invasion in ESCCs

**Figure 4:**
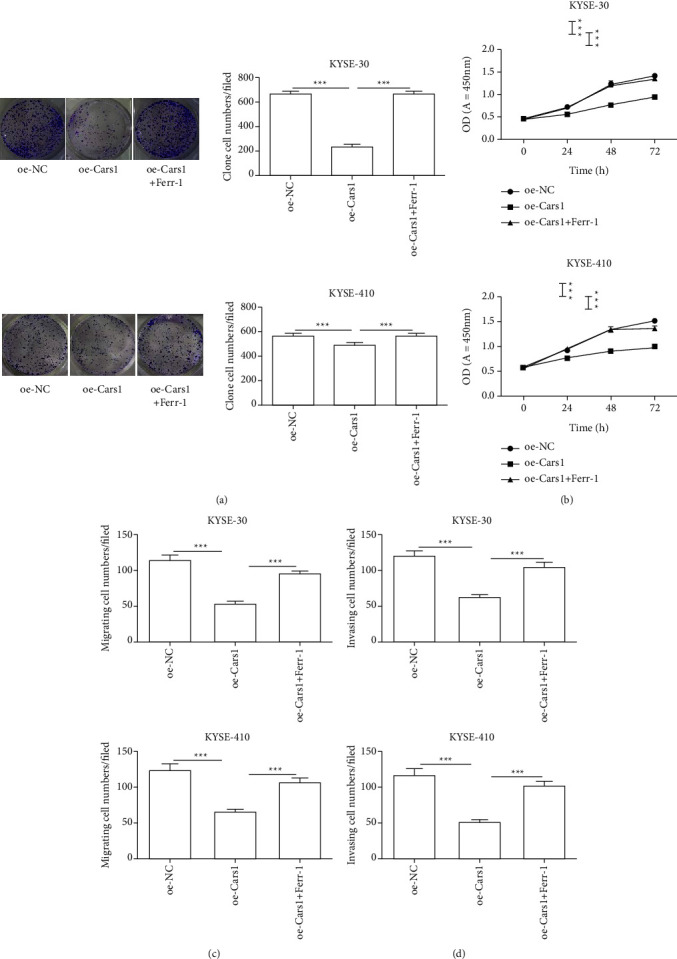
Effect of upregulated CARS1 and ferrostatin-1 on ESCC cell proliferation and the ability of migration and invasion. The colony formation assays (a) and the CCK-8 assays (b) were measured after being transfected with the CARS1 plasmid and treated with ferrostatin-1, respectively. The migration (c) and invasion (d) abilities in KYSE-30/410 cells after treatment have been examined by the transwell assay, respectively. ^*∗*^, ^*∗∗*^, ^*∗∗∗*^ indicate *P* < 0.05, 0.01, and 0.001, respectively.

To explore the function of CARS1 in the process of ferroptosis of ESCCs, the specific inhibitor ferrostatin-1 of ferroptosis was used in the study. The colony formation assay indicated that the combination group of upregulated CARS1 and ferrostatin-1 promoted the colony count in KYSE-30 and KYSE-410 as compared with the upregulated CARS1 group. Subsequently, the CCK-8 assay indicated a similar effect that the combination of upregulated CARS1 and ferrostatin-1 significantly increased proliferation at 24, 48, and 72 hours in ECSS cell lines as compared to the control group (Figure 4(a)). Therefore, it suggested that CARS1 was involved in the ferroptosis-induced cell death in ESCCs. Additionally, the ability of cell migration and invasion has been examined. It indicated that the combination group of upregulated CARS1 and ferrostatin-1 promoted both cell migration and invasion as compared to the upregulated CARS1 group (Figures 4(b) and 4(c)). It suggested that CARS1 participated in the migration and invasion of ESCCs caused by ferroptosis.

### 3.4. The Effects of Upregulated CARS1 and Ferrostatin-1 on Redox Reactions in ESCCs

**Figure 5:**
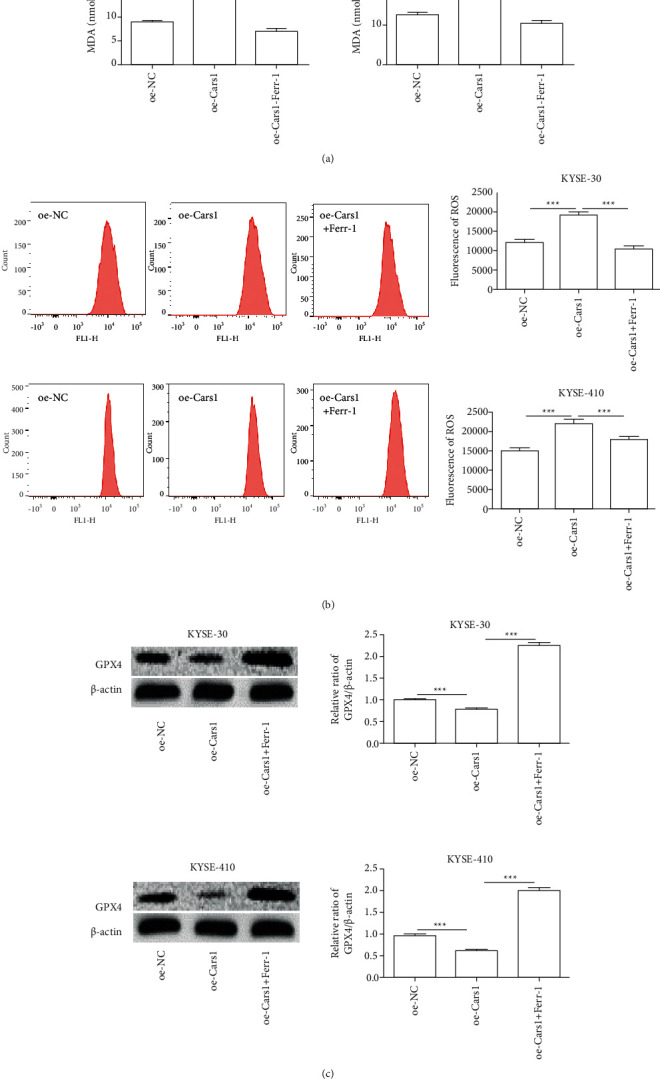
Effect of upregulated CARS1 and ferrostatin-1 on ESCC cell redox reactions. (a) The relative level of MDA was measured in KYSE-30 and KYSE-410 cells after transfecting with CARS1 plasmid and treated with ferrostatin-1. (b) The flow cytometry was conducted to check the ROS content of KYSE-30 and KYSE-410 cells after treatment. (c) Western blot was also detected. ^*∗*^, ^*∗∗*^, and ^*∗∗∗*^ indicate *P* < 0.05, 0.01, and 0.001, respectively.

To further investigate the combination of upregulated CARS1 and ferrostatin-1 on redox reactions in ESCCs, the relative level of MDA was first examined. It indicated that the combination of upregulated CARS1 and ferrostatin-1 decreased the content of MDA in KYSE-30 and KYSE-410 when compared to the upregulated CARS1 (Figure 5(a)). Subsequently, the flow cytometry experiment further examined that the combination of upregulated CARS1 and ferrostatin-1 significantly alleviated the cellular ROS content in KYSE-30 and KYSE-410 when compared to the control group (Figure 5(b)). Additionally, Western blot further exhibited that the combination of upregulated CARS1 and ferrostatin-1 significantly promoted GPX4 expression levels in KYSE-30 and KYSE-410 when compared to the upregulated CARs1 group (Figure 5(c)). Together, they suggested that CARS1 mainly alleviated the cellular redox reaction induced by ferroptosis in ESCCs.

### 3.5. The Effects of Downregulated CARS1 and Erastin on Proliferation, Migration, and Invasion in ESCCs

**Figure 6:**
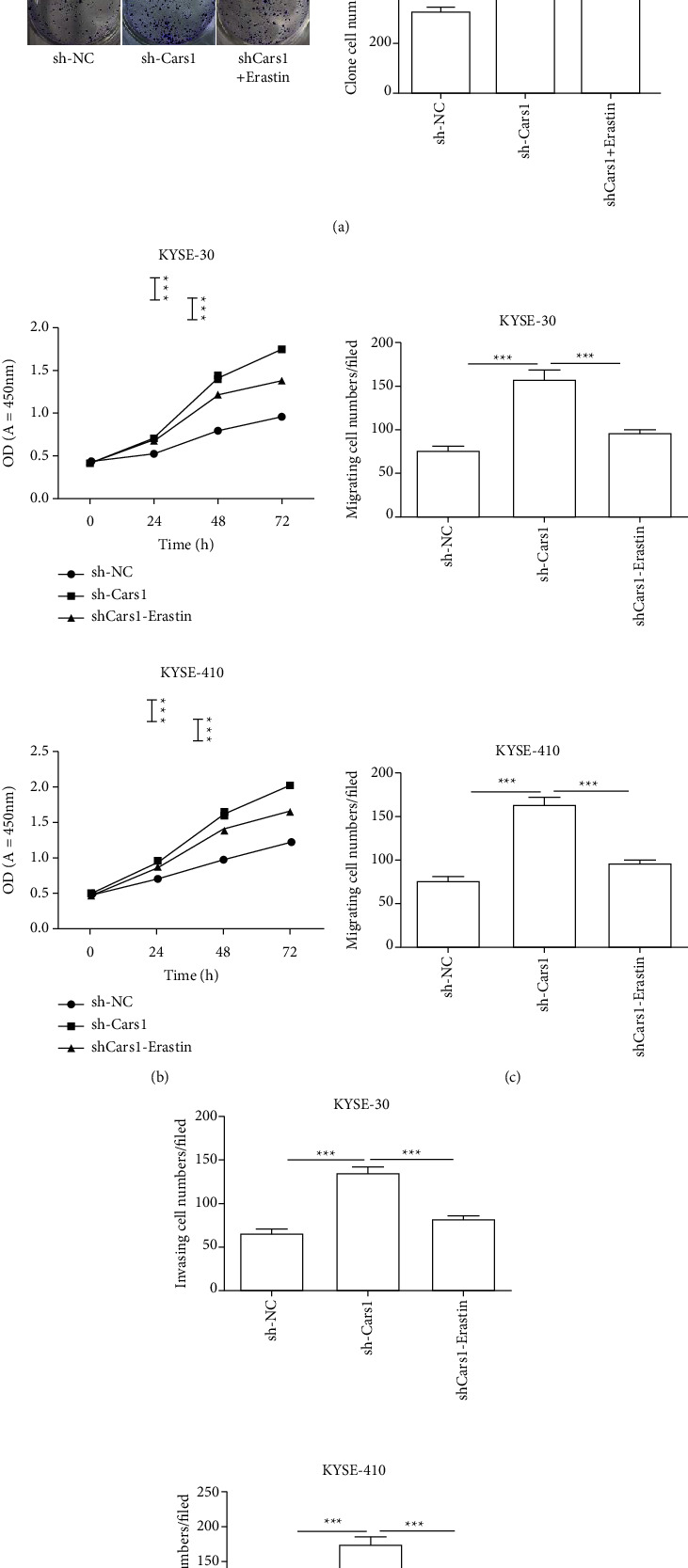
Effect of downregulated CARS1 and Erastin on ESCC cell. The colony formation assays (a) and the CCK-8 assays (b) were measured after being transfected with shCARS1 and Erastin with ferrostatin-1, respectively. The migration (c) and invasion (d) abilities in KYSE-30 and KYSE-410 cells after treatment have been detected by the transwell assay, respectively. ^*∗*^, ^*∗∗*^, and ^*∗∗∗*^ indicate *P* < 0.05, 0.01, and 0.001, respectively.

As an efficient ferroptosis inducer, Erastin could mediate ferroptosis-induced cell death via a series of small molecules, such as voltage-dependent anion channels. The colony formation assay indicated that the combination group of downregulated CARS1 and Erastin significantly decreased the colony count in KYSE-30 and KYSE-410 as compared with the downregulated CARS1 group. Subsequently, the CCK-8 assay indicated a similar effect that the combination of downregulated CARS1 and Erastin significantly increased the proliferation at 24 hrs, 48 hrs, and 72 hrs in KYSE-30 and KYSE-410 as compared to the control group (Figure 6(a)). Additionally, the transwell cell migration and invasion assay has been further examined. These results indicate that the combination group of downregulated CARS1 and Erastin decreased the ability of migration and invasion when compared to the downregulated CARS1 group (Figures 6(b) and 6(c)). The above results indicated that downregulated CARS1 suppressed cell proliferation and the ability of migration and invasion caused by ferroptosis in ESCCs.

### 3.6. The Effects of Downregulated CARS1 and Erastin on Redox Reaction in ESCCs

**Figure 7:**
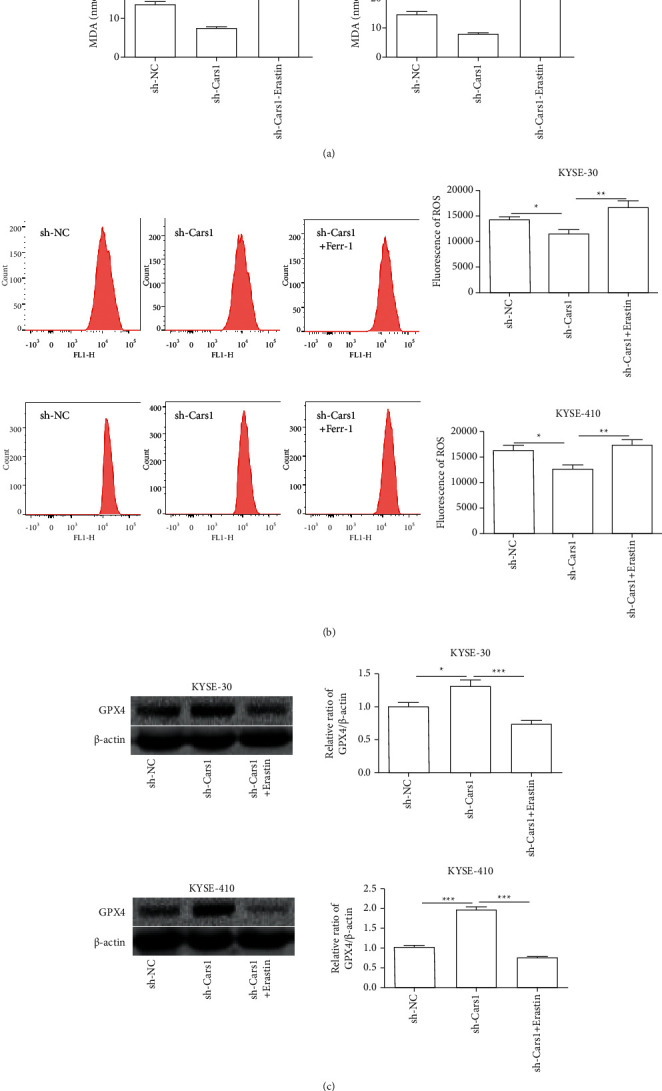
Effect of downregulation of CARS1 and Erastin on ESCC cell redox reaction. (a) The relative level of MDA is measured in KYSE-30/410 cells after being transfected with shCARS1 and treated with Erastin. (b) The flow cytometry is conducted to examine the content of ROS in KYSE-30/410 cells after treatment. (c) Western blot assay of KYSE-30/410 cells after treatment are detected. ^*∗*^, ^*∗∗*^, and ^*∗∗∗*^ indicate *P* < 0.05, 0.01, and 0.001, respectively.

To explore the combination of downregulated CARS1 and Erastin on redox reactions in ESCCs, we measured the relative level of MDA. The results showed that the combination of downregulated CARS1 and Erastin promoted the content of MDA in KYSE-30 and KYSE-410 when compared to the downregulated CARS1 (Figure 7(a)). Next, flow cytometry examined whether the combination of downregulated CARS1 and Erastin remarkably increased the cellular ROS content in KYSE-30 and KYSE-410 when compared to the control group (Figure 7(b)). Further, Western blot indicated that the combination of downregulated CARS1 and Erastin remarkably suppressed the relative protein level of GPX4 in KYSE-30 and KYSE-410 when compared with the downregulated CARS1 group (Figure 5(c)). When taken together, they suggest that the downregulation of CARS1 enhanced the sensitivity of Erastin-induced ferroptosis in ESCCs.

## 4. Discussion

ESCC is the most common malignancy of the digestive system in the world, and its mortality has always been in the forefront of malignant tumors. With the continuous improvement in diagnosis and treatment technology, the prognosis of ESCC patients has improved; however, the overall treatment effect is still not satisfactory. Early lymph node metastasis and postoperative local recurrence are important factors for ESCC patients' poor prognosis [10]. Therefore, it is very important to explore the new target of tumor metastasis mechanism for the therapy of ESCC treatment.

CARS1 contains a class 1 aminoacyl-tRNA synthetase, which is an essential enzyme catalyzing amino acids' ligation to their cognate tRNAs [11, 12]. Recent research indicated that CARS1 has prognostic values in predicting the overall survival of patients and targets ferroptosis as an alternative for therapy in several cancers [9, 13]. In the study, the experimental data indicated that CARS1 was remarkably upregulated in the ESCC tissues when compared to the normal esophageal mucosal tissue, suggesting that CARS1 is closely associated with patients' overall survival. Subsequently, we confirmed that the upregulation of CARS1 inhibited cell proliferation and the ability of migration and invasion. It implied that the upregulated CARS1 played a critical function in the progression of ESCC, which is consistent with the previous study [8].

Recent research has found that CARS1 is a new ferroptosis-related gene, predicting signature clinical prognosis in esophageal adenocarcinoma [8]. Our results showed that CARS1 participated in cell redox reaction could regulate the expression of GPX4, which is a key antioxidant enzyme that regulates iron death and protects cells from oxidative damage [14]. This process organizes the formation of iron-dependent toxic lipid reactive oxygen species and enables cells to resist iron death [15]. It suggested that CARS1 might have an antitumor function by promoting the process of ferroptosis.

Ferroptosis is a regulatory mode to cell death and lipid oxidation has a critical effect during the regulation process [16]. Previous studies have shown that GPX4 is a peroxidase of GSH and could inhibit ferroptosis-induced cell death. On the contrary, inhibiting GPX4 would enhance lipid peroxidation and trigger ferroptosis-induced cell death in the development and progression of tumor [17, 18]. Importantly, promoting the degradation of GPX4 could also promote this process [19, 20]. When ferroptosis occurs in cells, glutathione biosynthesis is inhibited [21]. To investigate whether CARS1 is associated with the ferroptosis-induced cell death, we conducted further experiments. After treatment with ferrostatin-1, a ferroptosis specific inhibitor, upregulated CARS1 remarkably enhanced the proliferation, migration and invasion in ESCC, diminished the MDA relative level and ROS content, and further enhanced GPX4 expression level. However, after treating cells with the ferroptosis inducer Erastin, downregulated CARS1 remarkably suppressed the proliferation, migration and invasion in ESCC, promoted MDA and ROS levels, and inhibited GPX4 expression level. When taken together, these results in the study suggest that CARS1 would promote the ferroptosis-induced cell death of ESCC.

This study confirmed the significant correlation between the relative level of CARS1 and the ESCC patients' survival rate. In this study, we further found a correlation between CARS1 and ferroptosis-induced cell death. The upregulation of CARS1 would promote ferroptosis-induced cell death, which suggests that CARS1 may become a new biomarker and potential therapeutic target for ESCC therapy.

## Data Availability

All data included in this study are available upon request with the corresponding author.

## Supplementary Materials

Supplementary Materials Figure S1 The effect of CARS1 expression after transfection in ESCC. (A) The relative level of CARS1 is measured using RT-PCR and Western blot after being transfected with the plasmid of CARS1 in KYSE-30/410. (B) The expression of CARS1 was measured using qRT-PCR and Western blot after being transfected with shCARS1 in KYSE-30 and KYSE-410. (^*∗∗*^*P* < 0.01 and ^*∗∗∗*^*P* < 0.001).

## References

[1] Abnet C. C., Arnold M., Wei W. Q. (2018). Epidemiology of esophageal squamous cell carcinoma. *Gastroenterology*.

[2] Huang J., Xu B., Mo H. (2018). Safety, activity, and biomarkers of SHR-1210, an anti-PD-1 antibody, for patients with advanced esophageal carcinoma. *Clinical Cancer Research*.

[3] Kim E. H., Shin D., Lee J., Jung A. R., Roh J. L. (2018). CISD2 inhibition overcomes resistance to sulfasalazine-induced ferroptotic cell death in head and neck cancer. *Cancer Letters*.

[4] Gupta G., Gliga A., Hedberg J. (2020). Cobalt nanoparticles trigger ferroptosis-like cell death (oxytosis) in neuronal cells: potential implications for neurodegenerative disease. *The FASEB Journal*.

[5] Song Z., Xiang X., Li J. (2020). Ruscogenin induces ferroptosis in pancreatic cancer cells. *Oncology Reports*.

[6] Dixon S. J., Lemberg K., Lamprecht M. (2012). Ferroptosis: an iron-dependent form of nonapoptotic cell death. *Cell*.

[7] Yang W. S., Stockwell B. R. (2008). Synthetic lethal screening identifies compounds activating iron-dependent, nonapoptotic cell death in oncogenic-RAS-harboring cancer cells. *Chemistry & Biology*.

[8] Zhu L., Yang F., Wang L. (2021). Identification the ferroptosis-related gene signature in patients with esophageal adenocarcinoma. *Cancer Cell International*.

[9] Hong Y., Lin M., Ou D., Huang Z., Shen P. (2021). A novel ferroptosis-related 12-gene signature predicts clinical prognosis and reveals immune relevancy in clear cell renal cell carcinoma. *BMC Cancer*.

[10] Martin J. C., Herbert B. S., Hocevar B. A. (2010). Disabled-2 downregulation promotes epithelial-to-mesenchymal transition. *British Journal of Cancer*.

[11] Kim S., You S., Hwang D. (2011). Aminoacyl-tRNA synthetases and tumorigenesis: more than housekeeping. *Nature Reviews Cancer*.

[12] Cho S., Kim S. B., Lee Y. (2020). Endogenous TLR2 ligand embedded in the catalytic region of human cysteinyl-tRNA synthetase 1. *J Immunother Cancer*.

[13] Liu H., Gao L., Xie T., Li J., Zhai T. s., Xu Y. (2021). Identification and validation of a prognostic signature for prostate cancer based on ferroptosis-related genes. *Frontiers in Oncology*.

[14] Agbor T. A., Demma Z., Mrsny R. J., Castillo A., Boll E. J., McCormick BA. (2014). The oxido-reductase enzyme glutathione peroxidase 4 (GPX4) governs SalmonellaTyphimurium-induced neutrophil transepithelial migration. *Cellular Microbiology*.

[15] Yang W. S., SriRamaratnam R., Welsch M. (2014). Regulation of ferroptotic cancer cell death by GPX4. *Cell*.

[16] Thandapani P. (2019). Super-enhancers in cancer. *Pharmacology & Therapeutics*.

[17] Whyte W. A., Orlando D., Hnisz D. (2013). Master transcription factors and mediator establish super-enhancers at key cell identity genes. *Cell*.

[18] Malhotra G. K., Yanala U., Ravipati A., Follet M., Vijayakumar M., Are C. (2017). Global trends in esophageal cancer. *Journal of Surgical Oncology*.

[19] Dukler N., Gulko B., Huang Y. F., Siepel A. (2017). Is a super-enhancer greater than the sum of its parts?. *Nature Genetics*.

[20] Tang F., Yang Z., Tan Y., Li Y. (2020). Super-enhancer function and its application in cancer targeted therapy. *Npj Precision Oncology*.

[21] Creyghton M. P., Cheng A. W., Welstead G. G. (2010). Histone H3K27ac separates active from poised enhancers and predicts developmental state. *Proceedings of the National Academy of Sciences of the United States of America*.

