# Supplementary figures and images for: Cysteinyl-tRNA Synthetase 1 Promotes Ferroptosis-Induced Cell Death via Regulating GPX4 Expression
Source: J Oncol. 2022 Sep 28;2022:4849174. doi: 10.1155/2022/4849174 (PMC9534673; doi:10.1155/2022/4849174)

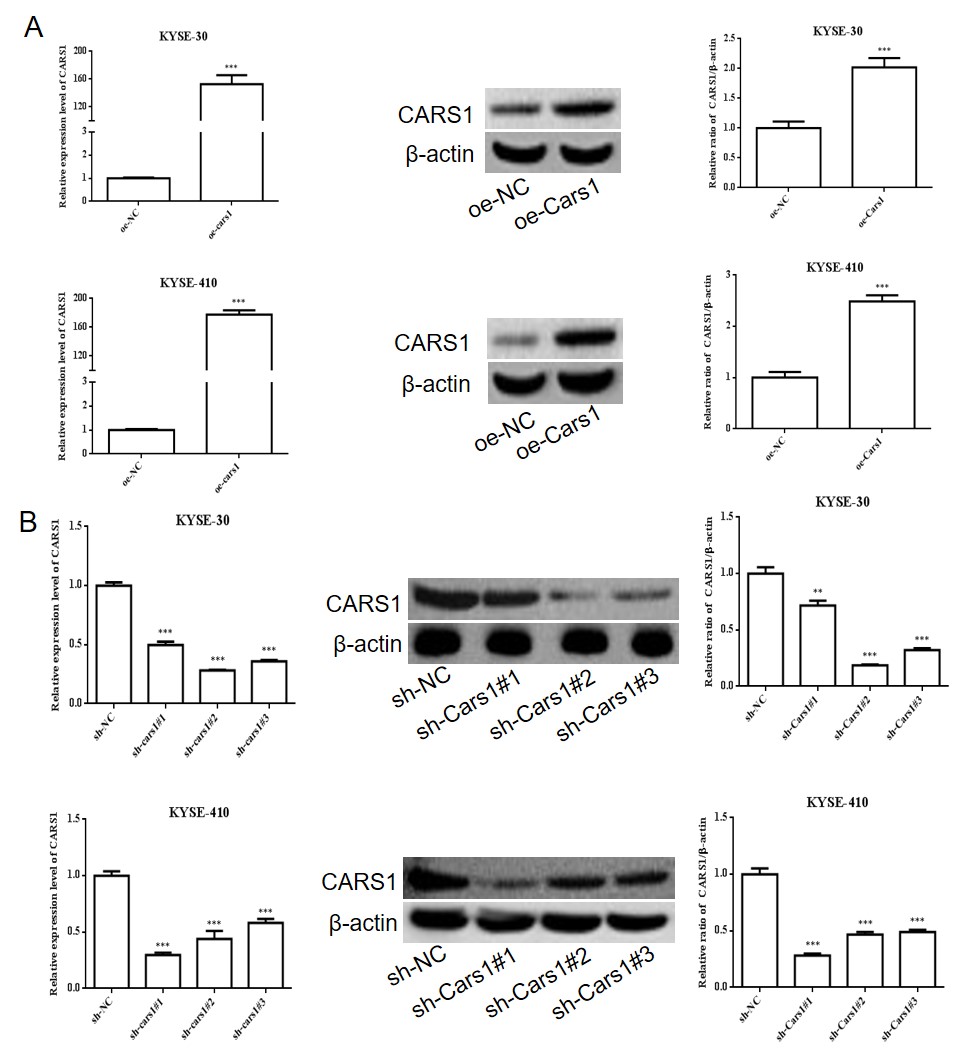

Supplement: Supplementary Materials — Figure S1 The effect of CARS1 expression after transfection in ESCC. (A) The relative level of CARS1 is measured using RT-PCR and Western blot after being transfected with the plasmid of CARS1 in KYSE-30/410. (B) The expression of CARS1 was measured using qRT-PCR and Western blot after being transfected with shCARS1 in KYSE-30 and KYSE-410. (∗∗P < 0.01 and ∗∗∗P < 0.001). [file 4849174.f1.jpg]
